# Novel TPP-riboswitch activators bypass metabolic enzyme dependency

**DOI:** 10.3389/fchem.2014.00053

**Published:** 2014-07-28

**Authors:** Christina E. Lünse, Fraser J. Scott, Colin J. Suckling, Günter Mayer

**Affiliations:** ^1^Life and Medical Sciences Institute, University of BonnBonn, Germany; ^2^Department of Pure and Applied Chemistry, University of StrathclydeGlasgow, UK

**Keywords:** riboswitches, triazolethiamine, click-chemistry, metal-chelating compounds, metabolic enzymes

## Abstract

Riboswitches are conserved regions within mRNA molecules that bind specific metabolites and regulate gene expression. TPP-riboswitches, which respond to thiamine pyrophosphate (TPP), are involved in the regulation of thiamine metabolism in numerous bacteria. As these regulatory RNAs are often modulating essential biosynthesis pathways they have become increasingly interesting as promising antibacterial targets. Here, we describe thiamine analogs containing a central 1,2,3-triazole group to induce repression of *thiM*-riboswitch dependent gene expression in different *E. coli* strains. Additionally, we show that compound activation is dependent on proteins involved in the metabolic pathways of thiamine uptake and synthesis. The most promising molecule, triazolethiamine (TT), shows concentration dependent reporter gene repression that is dependent on the presence of thiamine kinase ThiK, whereas the effect of pyrithiamine (PT), a known TPP-riboswitch modulator, is ThiK independent. We further show that this dependence can be bypassed by triazolethiamine-derivatives that bear phosphate-mimicking moieties. As triazolethiamine reveals superior activity compared to pyrithiamine, it represents a very promising starting point for developing novel antibacterial compounds that target TPP-riboswitches. Riboswitch-targeting compounds engage diverse endogenous mechanisms to attain *in vivo* activity. These findings are of importance for the understanding of compounds that require metabolic activation to achieve effective riboswitch modulation and they enable the design of novel compound generations that are independent of endogenous activation mechanisms.

## Introduction

**Figure 1:**
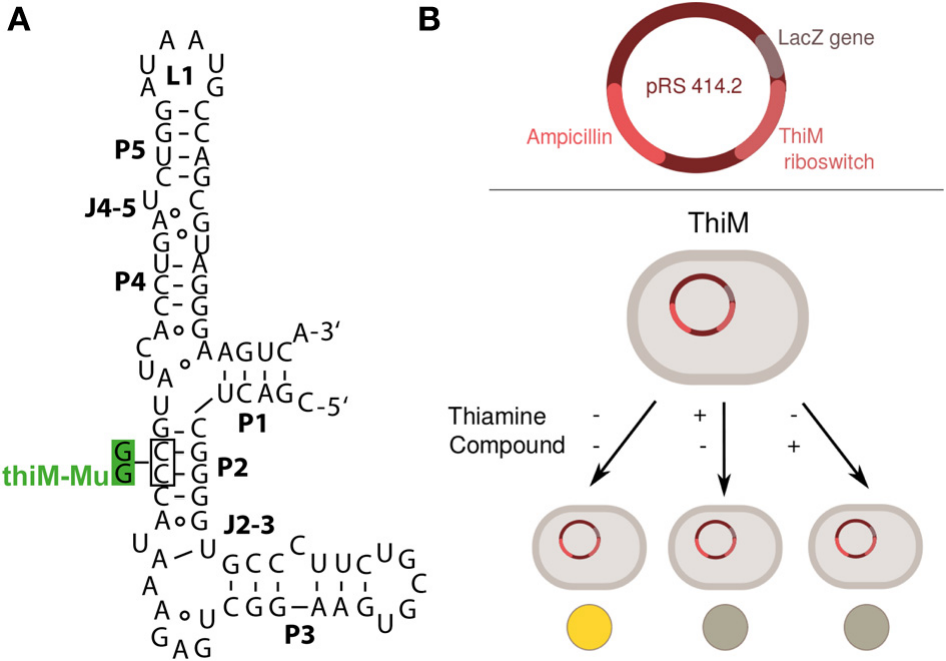
**(A)** Secondary structure of the aptamer domain of the *E. coli thiM* riboswitch and the non-binding *thiM* riboswitch variant (*thiM*-Mu), whose alterations in P2 are shown in green (Mayer et al., 2007). **(B)** Schematic drawing of plasmid constructs and screening set-up employed for evaluating effects of thiamine analogs (compounds) in DH5αZ1 cells or Keio collection strains. When thiamine is added, it is taken up by the bacteria and converted to thiamine pyrophosphate, which then acts on the *thiM* riboswitch causing a repression of reporter gene expression. Levels of the reporter gene β-galactosidase are assayed by cell lysis and reaction with *ortho*-nitrophenyl β-galactoside (ONPG) (see methods for detailed procedure).

**Figure 2:**
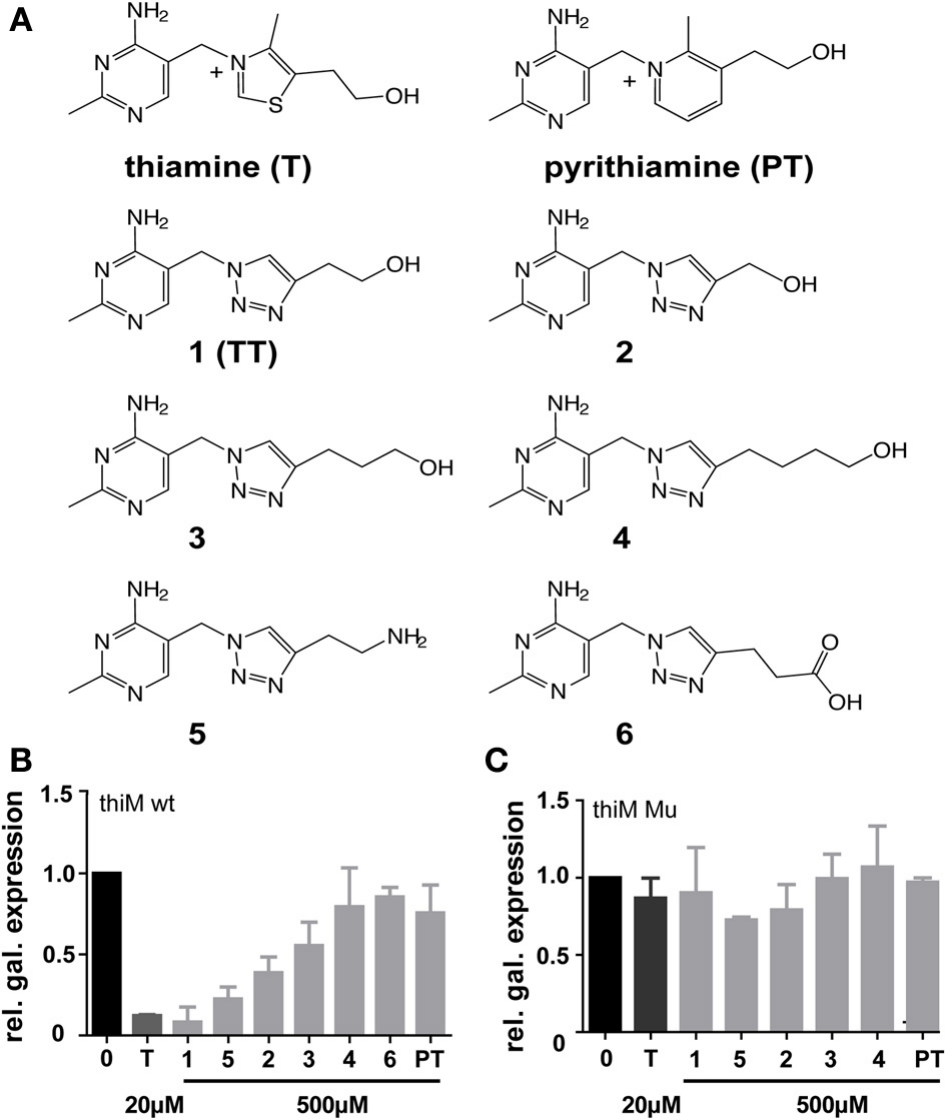
**(A)** Chemical structures of thiamine (T), pyrithiamine (PT) and compounds **1–6** that were assayed for influence on *thiM* riboswitch-dependent reporter gene expression and bacterial growth inhibition. **(B)** Relative β-galactosidase expression in the presence of either thiamine (20 μM) or compounds **1–6** and pyrithiamine **(PT)** (500 μM) in *E. coli* DH5αZ1 pRS414.2 *thiM* wt. Relative β-galactosidase expression in the presence of either thiamine (20 μM) or compounds **1–5** and pyrithiamine **(PT)** (500 μM) *E. coli* DH5αZ1 pRS414.2 *thiM*-Mu **(C)**. As compound **6** did not show significant effects in primary screenings **(B)** it was not used for further investigations **(C)**. Reporter gene activity is shown in relation to maximum β-galactosidase expression in DH5αZ1 bacteria (no addition of thiamine, black bars) and addition of 500 μM thiamine yield maximal reporter gene repression (dark gray bars).

Riboswitches are RNA elements mostly found in the 5′ UTR of bacterial mRNA that specifically sense the concentration of a small metabolite. Upon metabolite binding, substantial conformational changes occur that ultimately result in an on- or off-switch of gene expression. These regulatory elements represent a fundamental new means of controlling cellular processes in response to environmental conditions (Breaker, 2011). Riboswitches often regulate expression of essential genes and as such they are interesting target structures for the development of novel antibiotic compounds (Blount and Breaker, 2006). Artificial compounds acting successfully on riboswitches have to meet at least two criteria: (i) they must specifically bind to the relevant RNA structure and (ii) induce the conformational changes that finally lead to down- or up-regulation of gene expression. In recent years synthetic molecules that meet these criteria have been discovered for several riboswitch classes (Lünse et al., 2014). One of them is the TPP-riboswitch (Miranda-Rios et al., 2001; Winkler et al., 2002), which selectively interacts with thiamine pyrophosphate (TPP) by binding to its pyrimidine moiety through the so-called pyrimidine sensor helix (P2, J2-3, P3) of the aptamer domain. Conformational changes of the riboswitch resulting in repression of gene expression are only achieved if the pyrophosphate group of the ligand is recognized by the pyrophosphate sensor helix (P4, J4-5, P5, Figure 1A) (Edwards and Ferre-D'Amare, 2006; Serganov et al., 2006; Thore et al., 2006). While this riboswitch class has been shown to allow a greater degree of variation at the position of the thiazole ring, exemplified by the thiamine analog pyrithiamine (PT, Figure 2A), the presence of the pyrophosphate moiety is mandatory to interact with and, more importantly, to induce switching of the *E. coli thiM*-riboswitch (Winkler et al., 2002; Rentmeister et al., 2007). This necessity impedes the development of compounds that act on TPP-riboswitches, since either they cannot passively pass the cell wall due to the highly charged pyrophosphate moiety or they cannot efficiently activate the riboswitch if lacking this group. PT's *in vivo* activity most likely relies on hijacking endogenous metabolic enzymes that phosphorylate exogenously added PT, thereby yielding the active derivative pyrithiamine pyrophosphate (PTPP) (Iwashima et al., 1976). In this report we elaborate on a series of triazolethiamines (TT) that have been shown recently to act on TPP-riboswitches *in vitro*, if diphosphorylated yielding triazolethiamine pyrophosphate (TTPP). We investigated TT-activity in relation to the length and modification of the alkyl chain and the presence of endogenous proteins involved in thiamine metabolism. We found that the activity of triazolethiamine (TT) depends on the metabolic enzyme thiamine kinase (ThiK) and thiamine transporters. Most importantly, we demonstrated that ThiK dependency is bypassed by triazolethiamine-derivatives that bear phosphate mimicking and metal-ion chelating moieties. This strategy opens new avenues toward riboswitch activating compounds, in particular those that rely on phosphate groups to recognize and switch cognate RNA structures.

## Materials and methods

### β-galactosidase reporter gene assay

For β-galactosidase assays 5 ml pre-cultures of DH5αZ1, BW25113 or Keio deletion strains containing the appropriate plasmid constructs were prepared in LB Lennox standard medium (lysogeny broth Lennox, 10 g/l tryptone, 5 g/l yeast extract, 5 g/l NaCl in water) and incubated over night at 37°C and 155 rpm. Incubation was followed by photometric measurement of optical density at λ = 600 nm (OD_600_) and dilution of cells to an optical density of 0.5. This dilution was used to inoculate β-galactosidase expression cultures at a ratio of 1:500 in a final volume of 2 or 4 ml in M9 medium (5x M9 medium containing 15 g/l KH_2_PO_4_, 5 g/l NH_4_Cl, 2.5 g/l NaCl, 30 g/l Na_2_HPO_4_) containing final concentrations of 5 mM MgSO_4_, 0.2 wt% glucose, 0.2 μg/μl casamino acids (Difco), and 100 μg/ml ampicillin. Even though vitamin-deprived casamino acids were used, growth of thiamine auxotrophic strains was observed without addition of thiamine indicating that a small, but constant amount of thiamine must be present in the casamino acid stock. Minimal inhibitory concentration determinations in casamino acid free minimal medium with separately added amino acids revealed that at least 1 nM of thiamine is necessary to enable bacterial growth of the thiamine auxotrophs investigated. The expression cultures contained either 20 or 500 μM of thiamine (Sigma), 500 μM pyrithiamine (Sigma) or 500 μM of the compounds, respectively. Controls received neither thiamine, or pyrithiamine nor compounds. If compounds had to be dissolved in DMSO, controls were supplemented with equal amounts (final DMSO concentration: 1%). The cultures were incubated for 24 h at 37°C and 150 rpm. After 24 h incubation the optical density was measured. The cells were centrifuged at 4500 g for 5 min and the pellets were washed twice in 400 μl 1x PBS (137 mM NaCl, 2.7 mM KCl, 10 mM Na_2_HPO_4_x2H_2_O, 2 mM KH_2_PO_4_, pH 7.4). The pellets were resuspended in 200 μl 1x lysis buffer (Reporter Lysis Buffer, Promega), incubated for 15 min at room temperature and cells were pelleted. Supernatants were used for all following steps. 75 μl of the respective lysates were mixed with 75 μl of 2x assay buffer (200 mM sodium phosphate buffer pH 7.3, 2 mM MgCl_2_, 100 mM β-mercaptoethanol, 1.33 mg/ml ONPG) and incubated for 5-15 min at 37°C. The reaction was stopped by the addition of 250 μl 1 M Na_2_CO_3_ and absorbance at 420 nm was measured immediately using a Nanoquant (Infinite 200, Tecan). Reporter gene assays were performed in clear, flat bottom 96-well plates. Miller units were calculated according to the following formula: Miller units = (1000*OD420)/(OD 600*incubation time [min]*culture volume [L]) and Miller units of controls without thiamine or compound added were set to 1. Samples were run in duplicate and experiments were repeated at least three times.

## Results

**Scheme 1:**
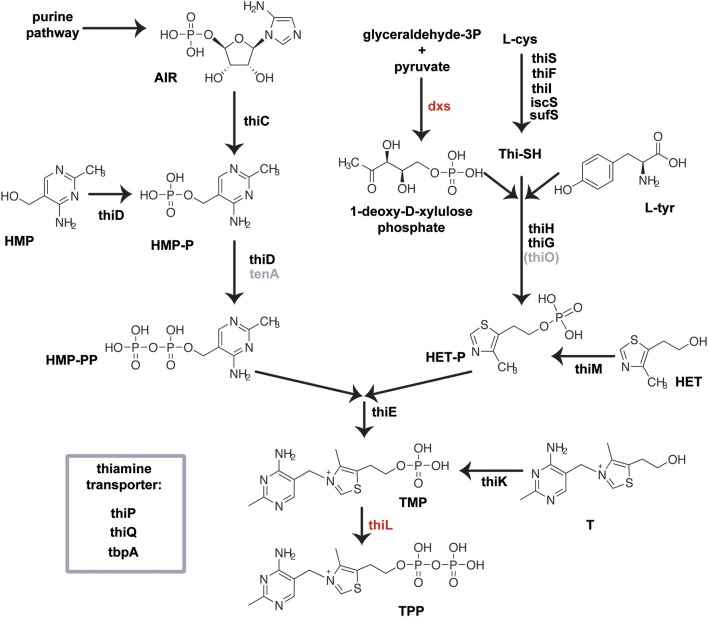
**Biosynthesis of TPP in *E. coli* is accomplished by the separate synthesis of the pyrimidine and thiazole moiety, which are finally coupled and phosphorylated to form TPP (Begley et al., 1999)**. The biosynthesis of the pyrimidine part of thiamine starts with an intermediate of the purine biosynthesis pathway, namely aminoimidazole ribotide (AIR). The thiamine biosynthesis protein ThiC whose exact function remains to be elucidated, converts AIR to hydroxymethylpyrimidine-phosphate (HMP-P), which is subsequently phosphorylated by the bifunctional HMP/ HMP-P kinase ThiD to yield hydroxymethyl-pyrimidine pyrophosphate (HMP-PP). The thiazole moiety of thiamine is derived from tyrosine, cysteine and 1-deoxy-D-xylulose phosphate (DXP). In a yet unresolved chain of reactions featuring thiF, thiS, thiG, thiH and thiI gene products, hydroxyethyl-thiazole phosphate (HET-P) is formed. HMP-PP and HET-P are joined by one enzymatic step mediated by the ThiE protein, followed by phosphorylation of the formed TMP by ThiL to create TPP. Three distinct kinases, ThiM, ThiD, and ThiK, are involved in the salvage of HET, HMP, and thiamine, respectively, from the culture medium. Thiamine, thiamine phosphate, and thiamine pyrophosphate are actively transported in enteric bacteria using the ABC transport system ThiBPQ (Webb et al., 1998). At present, no other distinct thiamine transporters, neither HET nor HMP transport systems, have been identified in bacteria (Rodionov et al., 2002). Essential genes are colored red, gray genes are not validated in *E. coli*.

Thiamine analogs that bear a 1,2,3-triazole group instead of the thiazole heterocyclic moiety as found in thiamine were synthesized and investigated for TPP-riboswitch activation using a β-galactosidase reporter gene assay (Figure 1B). In this assay the TPP-riboswitch *thiM* from *E. coli* was cloned into the 5′-UTR of β-galactosidase. The DH5αZ1 *E. coli* strain was transformed by this plasmid (Simons et al., 1987; Diederich et al., 1992; Lutz and Bujard, 1997). Due to the thi-1 mutation, DH5αZ1 cells are thiamine auxotroph, which means they are dependent on thiamine uptake from the growth medium (Supplementary Table 1). This enables direct external control of the availability of this vitamin. Synthesis of TT-compounds was achieved using a “click” chemistry approach utilizing a common azide intermediate, which was then further reacted with the appropriate substituted alkyne yielding corresponding thiamine analogs (Figure 2A, Supplementary Figure 1). Subsequent monitoring of β-galactosidase activity was performed in the presence and absence of thiamine or triazolethiamine (TT) derivatives. Addition of thiamine to the *E. coli* cultures resulted in a complete loss of β-galactosidase expression; an IC_50_-value of 0.015 μM was observed (Figure 2 and Table 1). In line with previous findings, pyrithiamine (PT) was shown incapable of β-galactosidase repression in DH5αZ1 (Figure 2B) (Sudarsan et al., 2005). On the other hand, the addition of 500 μM TT (**1**) represses the expression of β–galactosidase expression to a level similar to that of thiamine. TT has been shown to depend on diphosphorylation to gain activity *in vitro* (Chen et al., 2012). Thus, we anticipate that TT is phosphorylated by the endogenous set of thiamine converting enzymes. Shortening (**2**) or elongating the alkyl side chain (**3** and **4**, Figure 1A) results in a loss of repression with increasing deviation from the natural alkyl chain length (Figure 1B). This is most likely due to reduced uptake and/or phosphorylation of these compounds, but could also be because of a poor fit of phosphorylated triazolethiamines to the riboswitch. Previously, it has been shown that the *thi*-box aptamer domain is somewhat adjustable to ligand size through compaction of pyrimidine and phosphate sensor helices (Edwards and Ferre-D'Amare, 2006). However, this low affinity ligand binding [thiamine *K_D_* = 50 μM vs. TPP *K_D_* = 50 nM, (Sudarsan et al., 2005)] is unlikely to efficiently induce subsequent changes in the expression platform (Serganov et al., 2006) and thus reporter gene expression. Interestingly, compound **5**, which bears an amino group instead of a hydroxyl residue on the ethyl-alkyl side chain of TT, also represses reporter gene activity. How this compound would be activated through *in vivo* phosphorylation is unclear. However, it is possible that **5** is also phosphorylated in the same way as TT. Experiments using a mutant version of the *thiM* riboswitch (*thiM*-Mu, Figure 1A), which is incapable of binding to TPP showed that neither of the investigated TT-compounds represses gene expression in these assays. These data underline that repression of gene expression truly relies on riboswitch binding and activation. We next measured IC_50_-values of these compounds, to evaluate concentration dependent repression of gene expression. TT (**1**) revealed the strongest effect with an IC_50_-value of 8.4 μM (Table 1 and Supplementary Figure 2). Compounds with increasing alkyl-chain length (**3** and **4**) showed no concentration dependent inhibition curves (Table 1) and, as expected for this bacterial strain, PT did not show an effect on β-galactosidase expression (Table 1, Figure 2A). In order to investigate the dependence of **TT** activity on endogenous proteins and enzymes involved in thiamine biosynthesis, several strains of the Keio collection were investigated, that all originate from the strain BW25113, and contain deletions of non-essential genes known to be involved in thiamine metabolism of *E. coli* (Scheme 1) (Baba et al., 2006).

**Table 1:** **IC_50_-values of thiamine, pyrithiamine and compound 1–5 for inhibition of reporter gene expression in DH5αZ1 cells are listed**.

**Compound**	**IC_50_ (μM) (DH5αZ1)**	**95% Confidence interval**
Thiamine	0.015	0.012–0.019
1 (TT)	8.4	4.6–15.6
2	43.3	13.4–140
3	n.a.	n.a.
4	n.a.	n.a.
5	22.9	12.1–43.4
PT	n.a.	n.a.

*Means and 95% confidence intervals of at least three independent experiments, measured in duplicates, are shown. n.a., not applicable; where data points could not be fitted to a dose-response curve. TT, Triazolethiamine; PT, Pyrithiamine*.

**Figure 3:**
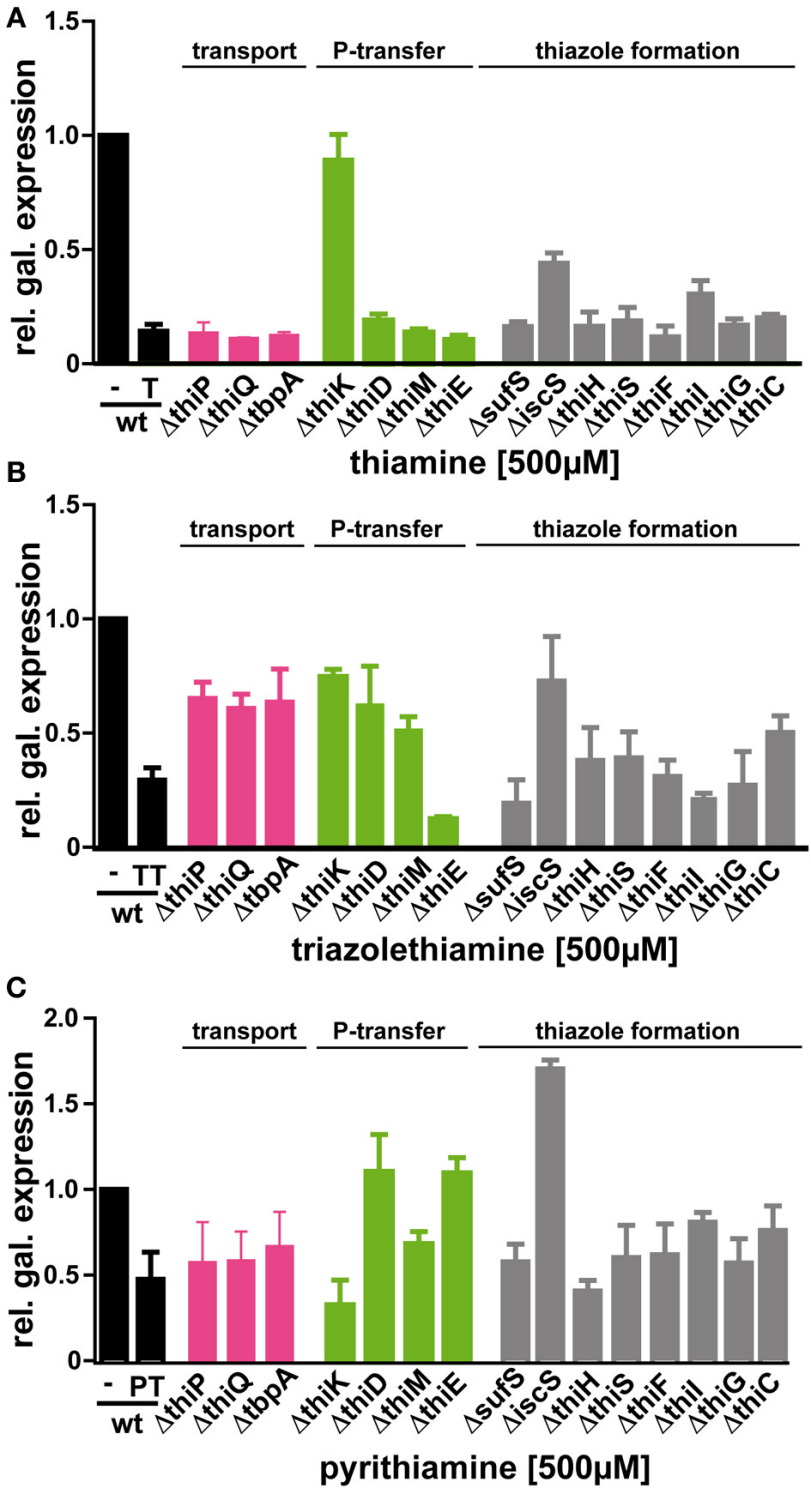
**Effect of thiamine (A), triazolethiamine (B) or pyrithiamine (C) on reporter gene expression in BW25113 (black bars) and BW25113 strains with the indicated deletion**. Minimal medium was supplemented with 500μM of thiamine (**T**), **TT** or **PT**. magenta bars: thiamine transport genes; green bars: phosphorylation enzymes; gray bars: genes involved in thiazole formation.

**Figure 4:**
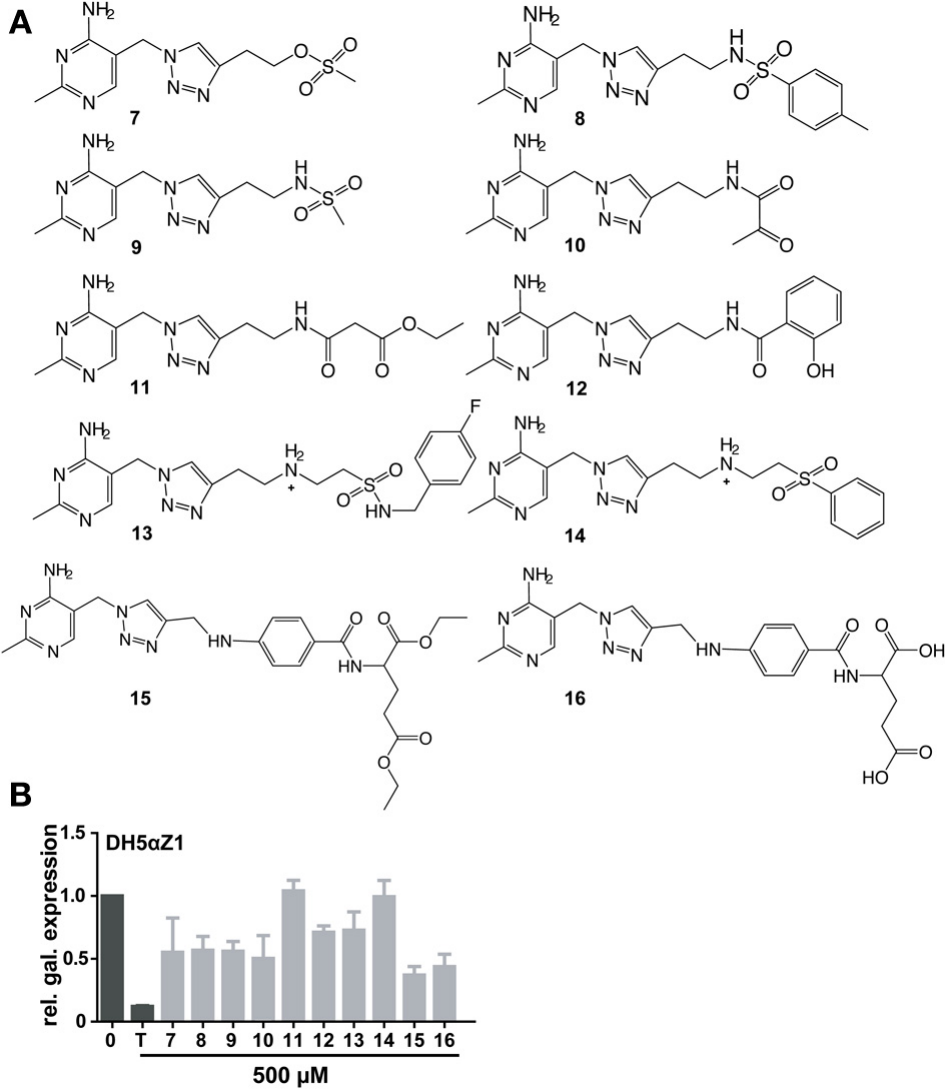
**Chemical formulas of triazole thiamine analogs with phosphate mimicking groups (A)**. Relative β-galactosidase expression in the presence of thiamine or compound (500μM) in DH5αZ1 cells is shown **(B)**.

Thiamine is synthesized from two precursors, hydroxy/-methyl/-pyrimidine diphosphate (HMP-PP) and hydroxyethylthiazole phosphate (HET-P), which are produced independently and finally joined to form thiamine phosphate (Begley et al., 1999). Further phosphorylation yields the cofactor TPP (Scheme 1). The Keio deletion strains were transformed with the β-galactosidase reporter plasmid containing either the wild type or the mutant TPP-riboswitch and effects of thiamine, **TT** or **PT** on reporter gene expression were assayed (Table 2, Figure 3, Supplementary Figures 9, 10). In most strains the addition of thiamine led to a strong decrease in β-galactosidase expression that is comparable to that observed for the wild type strain. As expected, the *thiK* deletion strain (Δ*thiK*) revealed decreased thiamine sensitivity, since this enzyme is required for the phosphorylation of thiamine to TPP. When adding thiamine, *thiI* and *iscS* deletion strains showed slightly increased levels of reporter gene expression indicating a loss of TPP-riboswitch activation (Figure 3A). Investigating the impact of **TT** (Figure 3B) on TPP-riboswitch dependent reporter gene expression in the deletion strains revealed a prominent increase in reporter gene expression for those strains containing deletions of thiamine transport genes (Scheme 1; *thiP*, *thiQ*, *tbpA*) in comparison to wild type cells (Figure 3B, magenta bars). This may indicate that **TT** uptake is, at least in part, mediated by these ABC-transporters. However in the presence of **PT**, reporter gene expression in these strains was scarcely increased in comparison to the wild type strain (Figure 3C). This suggests that **PT**, just like thiamine (Figure 3A), must use other routes of cell entry apart from the thiamine-specific active transporter. Possibly, its positive charge supports passive diffusion processes, as believed to occur for thiamine as well (Webb et al., 1998). Besides the dependence on transport proteins, differential effects on several other deletion strains involved in phosphate-transfer or thiazole formation were observed (Figure 3, green and gray bars, respectively). Assaying Δ*thiK*, Δ*thiD*, and Δ*thiM*, strains, whose deleted genes are involved in phosphate transfer reactions, revealed increased β-galactosidase expression in the presence of **TT** compared to wild type, whereas Δ*thiE* showed decreased β-galactosidase expression (Figure 3B). As the deletion of *thiE* renders the bacteria incapable of synthesizing any TPP from its HMP and HET precursors (Scheme 1), the reporter gene expression is mainly influenced by external thiamine or compound supply. Interestingly, the prominent decrease of β-galactosidase expression in the presence of **TT** is comparable to that found for thiamine at the same concentration in the wild type strain BW25113 (Figure 3A, black bars). In Δ*thiE* cells, lower intracellular concentrations of thiamine compete for any possible enzymatic **TT**-activation mechanism (such as phosphorylation or take-up). Therefore, the same amounts of **TT** lead to a stronger decrease of reporter gene expression in these cells in comparison to other deletion strains (Figure 3B). In the presence of **TT**, *thiC* and *iscS* deletion strains which are involved in formation of the thiazole moiety of thiamine revealed increased β-galactosidase expression (Figure 3B). At the same time β-galactosidase expression in *thiI*, *thiG*, *thiH* and *sufS* deletion strains remained unaffected or was slightly decreased in the presence of **PT** (Figure 3C). Finally, PT-treated *E. coli* strains containing deletions of the genes *thiD* and *thiC*, which are involved in the biosynthesis of the pyrimidine-containing precursor HMP-PP (Scheme 1), show an increase in reporter gene expression (Figure 3C). The same is observed for the Δ*thiE* strain. The deletion of *iscS* renders bacteria less susceptible to thiamine, **TT** or **PT** (Supplementary Figure 3). However, it should be noted that the Δ*iscS* strain showed slower growth than any of the other deletion strains investigated. This reduced fitness may be due to the fact that ThiI and IscS are not only involved in thiamine metabolism but also needed in the 4-thiouridine biosynthetic pathway (Scheme 1). Interestingly, deleting the thiamine kinase ThiK abolished thiamine and **TT** dependent inhibition of reporter gene expression (Figures 3A,B). This finding underlines the hypothesis that **TT** is only active *in vivo* in its phosphorylated form. Due to its close structural similarity to the natural substrate thiamine, **TT** is likely to be recognized and phosphorylated by endogenous bacterial enzymes such as ThiK. Using ITC measurements Chen and colleagues revealed that the chemically synthesized, diphosphorylated form of **TT**, named triazolethiamine pyrophosphate (TTPP), binds to the *thiM* (Figure 4A) riboswitch with an affinity of 370 nM, whereas for the natural ligand TPP a K_D_ of 8 nM was measured (Chen et al., 2012). Additionally, *in vitro* translation assays proved that binding of TTPP induces a change in riboswitch secondary structure to sequester the SD-sequence and inhibits efficient protein translation (Chen et al., 2012). Therefore, endogenous diphosphorylation of **TT** would generate a very potent TPP-riboswitch activator. Hence, we were intrigued to find that reporter gene expression for the *thiK* deletion strain is dramatically decreased in the presence of **PT** (Figure 3C). This suggests that PT either does not need to be activated by pyrophosphorylation through ThiK, that it can also be phosphorylated by other endogenous enzymes like ThiE or that it is converted into TPP through salvage pathways.

**Table 2:** **IC_50_-values of thiamine for inhibition of reporter gene expression in *E. coli* containing the indicated deletion of a non-essential thiamine biosynthesis gene are shown as mean and 95% confidence interval of at least three independent experiments, measured in duplicates**.

**Strain**	**IC_50_ (μM)**	**95% Confidence Interval**
BW25113 (wt)	0.032	0.022–0.047
ΔthiP	0.93	0.74–1.2
ΔthiQ	1.5	1.0–2.1
ΔtbpA	1.3	0.97–1.7
ΔthiL	0.027	0.018–0.039
ΔthiK	n.a.	n.a.
ΔsufS	0.025	0.018–0.036
ΔthiD	0.019	0.012–0.030
ΔthiM	0.016	0.010–0.026
ΔiscS	n.a.	n.a.
ΔthiH	0.028	0.021–0.037
ΔthiS	0.033	0.018–0.061
ΔthiF	0.009	0.0062–0.013
ΔthiE	0.071	0.045–0.11
ΔthiC	0.023	0.015–0.035
ΔthiG	0.0091	0.0054–0.015

*n.a., not applicable, where data points could not be fitted to a dose-response curve*.

*Respective curves are shown in Supplementary Figures 9, 10*.

**Figure 5:**
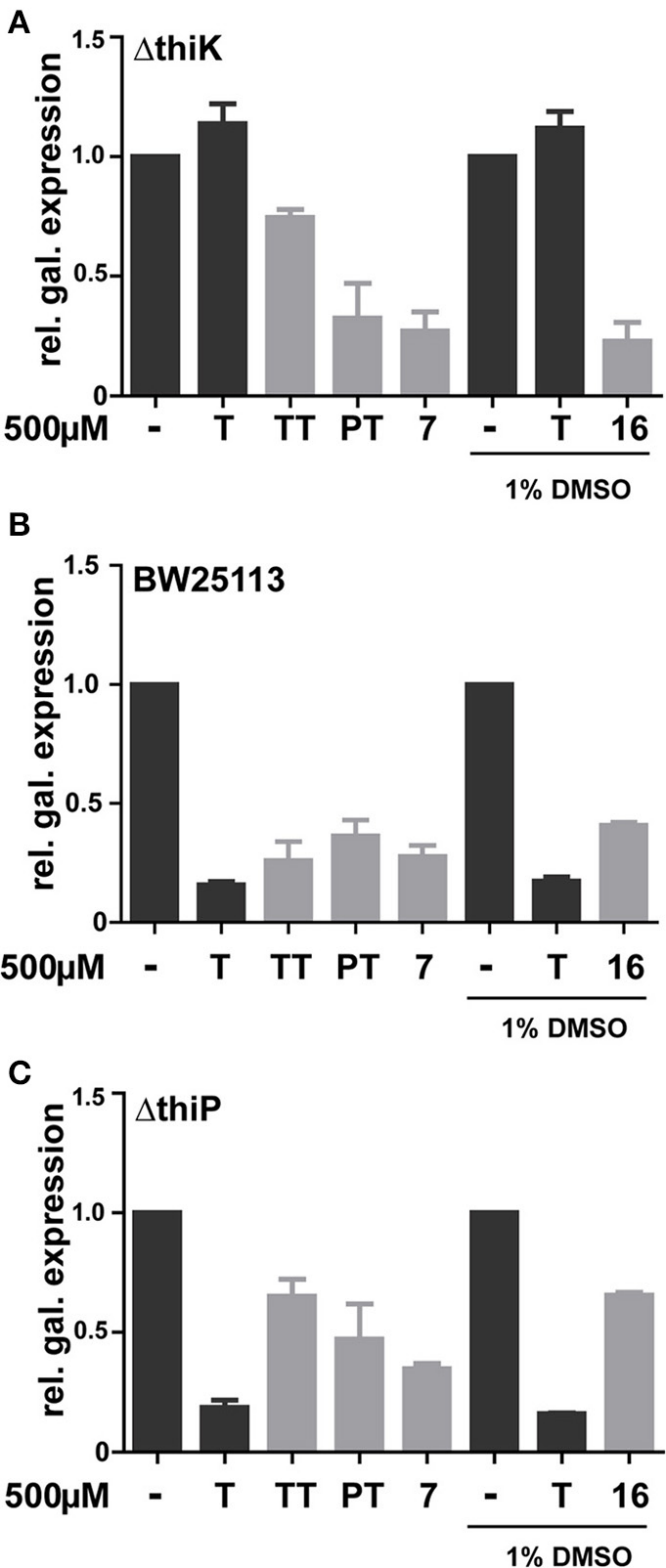
**Effect of thiamine, TT, PT and phosphate mimics 7 and 16 on β-galactosidase expression in *E. coli* strains Δ*thiK* (A), BW25113 (B), and Δ*thiP* (C)**. Minimal medium (refer to experimental procedures for details) was supplemented with 500μM of analyte. Compound **16** was dissolved to a stock solution of 100mM in 100% DMSO, assay controls also contained a final DMSO concentration of 1%.

In order to bypass ThiK-dependency of **TT** activity in *E. coli*, triazolethiamine derivatives that were intended to be independent of endogenous phosphorylation were synthesized and their impact on riboswitch regulation of β-galactosidase expression was studied. These compounds contain a number of phosphate mimics (sulfate, sulfonamide, and sulfone) and metal-chelating groups (dicarbonyl compounds) that might be expected to interact with phosphate-binding metal ions in proteins (Supplementary Figures 4–8). A “click” chemistry approach was used to generate sulfone **7** and the methotrexate- or folate- like compounds **15** and **16** (Figure 4A). Amide couplings were performed to obtain the sulfonamides **8** and **9** and also to obtain the pyruvate, malonate and salicylic acid containing compounds **10**, **11**, and **12**, respectively (Figure 4A) (Erixon et al., 2008). Additionally, a Michael addition was used to generate the sulfones **13** and **14**. With respect to the phosphate mimics, the sulfonamide **13** and the sulfone **14** that are connected to a benzyl group scarcely activate the *thiM* riboswitch in DH5αZ1 cells. The sulfonamide **8** had a similar effect on β-galactosidase expression in the thiamine auxotroph strain. Interestingly, compounds containing the planar benzene ring are not as active in *thiM* riboswitch activation, even though it is imaginable that this aromatic ring could tighten compound-riboswitch interaction by π-π-stacking with RNA nucleobases (Edwards and Ferre-D'Amare, 2006; Thore et al., 2006). Compounds **15** and **16** were found to be the most potent repressors of reporter gene expression (Figure 4B, ~0.5 fold β-galactosidase expression). Compounds of this group have enough flexibility in principle to interact with two metal ions via their ester or carboxyl groups. Interestingly, these molecules activate the *thiM* riboswitch extensively, despite their relatively large size. The carboxylate ethyl esters like **15** are likely to enter the cell passively and are probably hydrolyzed by esterases resulting in **16**. To investigate whether the most potent phosphate mimics exhibit a ThiK independent reduction in reporter gene expression, compounds **7** and **16** were chosen and assayed in the thiamine kinase deficient *E. coli* strain (Figure 5A). In contrast to addition of thiamine or **TT**, which do not reduce reporter gene expression, the addition of the same amount of **7** and **16** induces a decrease of β-galactosidase expression to approximately 0.25 fold (Figure 5A). However, Δ*thiK* strains transformed by the mutated *thiM* riboswitch construct do not or only slightly respond to thiamine or compound addition (Supplementary Figure 11A). In wild type *E. coli* strain BW25113 thiamine has the strongest influence on β-galactosidase repression. **TT** and compound **7** are almost as efficient in inhibiting gene expression whereas **PT** and compound **16** show somewhat weaker effects (Figure 5B). Again no significant influence on reporter gene expression in cells containing the mutant *thiM* riboswitch was observed (Supplementary Figure 11C). Investigating the deletion of one subunit of the active thiamine transporter (ThiP) revealed increased levels of reporter protein for **TT**, **7**, and **16**, indicating that these molecules are at least in part taken-up via this mechanism (Figure 5C). The investigation of the mutant riboswitch construct *thiM*-Mu proves these compound effects to be specific for *thiM* riboswitch activation, as the mutated RNA does not alter protein expression (Supplementary Figure 11).

## Discussion

TPP riboswitch are interesting and attractive target structures for developing antibacterial compounds. They form the most extensive riboswitch class with representatives found in bacteria, archea and plants (Kubodera et al., 2003; Sudarsan et al., 2003; Bocobza et al., 2007). Here we show that replacing the thiazole heterocycle with 1,2,3-triazole is a valuable strategy to generate thiamine analogs that interact with TPP riboswitches and, thus, induce repression of gene expression. These findings are in line with the observations of Chen al. recently reporting that the diphosphorylated triazolethiamine indeed interacts with the *thiM* riboswitch *in vitro*, albeit with decreased affinity (Chen et al., 2012). We went a significant step further and demonstrated that these compounds are effective in *E. coli* and, more importantly, that their activity depends on proteins involved in the metabolic pathways of thiamine uptake and synthesis.

The data shown above indicate that activity of **TT** depends on active uptake and endogenous phosphorylation by ThiK. Surprisingly, **PT** was found to be independent of ThiK and transporters. Therefore, **PT** is either phosphorylated by other kinases apart from ThiK or its phosphorylation, against all previous hypotheses, is not necessary for *in vivo* activity (Edwards and Ferre-D'Amare, 2006; Warner et al., 2014). Unphosphorylated **PT** is known to have a more than 1000 fold lower affinity for the TPP-riboswitch, whereas diphosphorylation results in a K_D_ that is only 3fold higher than that of TPP (Sudarsan et al., 2005). Although **PT** was shown to bind to the pyrimidine sensor helix of the aptamer domain by one hydrogen bond and the stacking of the aminopyrimidine ring, crystal structures revealed that two hydrogen bonds have been lost in comparison to TMP or TPP interactions. The pyrophosphate sensor helix is largely disordered and it is questionable whether this riboswitch conformation allows gene regulation upon **PT** binding (Edwards and Ferre-D'Amare, 2006). Furthermore, the increase in *thiM*-riboswitch dependent reporter gene expression in the presence of **PT** in Δ*thiD* or Δ*thiC* strains suggests the existence of a **PT**-detoxification mechanism in *E. coli* as proposed for *B. subtilis* by Sudarsan et al. (2005). If indeed there was a thiaminase-II-like enzyme present in *E. coli*, HMP could be recovered from **PT** and used for the generation of thiamine through combination with *de novo* synthesized thiazole (Scheme 1). This **PT** recycling would lead to a decrease of its intracellular concentration, hence a diminished influence on the TPP-riboswitch-dependent gene expression. At the same time, the newly generated TPP is likely to be used immediately for its biological purpose without accumulation and hence a need for riboswitch-mediated down regulation of gene expression. Even though a TenA-like protein has not yet been identified in *E. coli*, *in silico* predictions indicate that ThiC may represent a functional equivalent of TenA (Morett et al., 2003).

We further show that equipping **TT** with metal-chelating groups results in ThiK- and transport-independent activity. This indirectly proves that **TT** requires phosphorylation and indicates a possible route in riboswitch activator design which circumvents the necessity of endogenous proteins and renders riboswitch inhibitors independent of bacterial pathways. The most promising phosphate-mimics were tested for their dependence on ThiK or active thiamine transport mechanisms. Whereas activity of compound **16** appears to be ThiK-independent, its transport is strongly influenced by ABC transporter integrity. At the same time, effects of compound **16** in wild type cells are not as prominent as those observed for **TT**. Compound **7** however, does not only reveal strong reporter gene repression, but this effect is even independent of ThiK or ThiP presence. Hence, future compound designs should take characteristics of compound **7** into account in order to provide novel chemical entities whose *in vivo* activity does not depend on the individual enzyme repertoire of the target cell. Also, investigating potential routes of metabolism of phosphate-mimicking compounds may unveil further information and support the generation of even more precise riboswitch-regulating compounds.

The distinct efficiencies of **TT**-derivatives and **PT** in repression of gene expression, further point at possible secondary effects of the employed metabolite analogs. These side effects may rely on interactions with enzymes involved in thiamine pyrophosphate metabolic pathways. Indeed it has been shown that triazole-derivatives of thiamine pyrophosphate inhibit enzymes involved in the biosynthesis of TPP *in vitro* (Erixon and Dabalos, 2007).

Others have shown that analogs with a positively charged middle ring bind much better to the *thiM* riboswitch than ones with a neutral ring at this position (Chen et al., 2012). It was suggested that this is due to the electron drawing effect of the middle ring, which enhances the binding of the pyrimidine moiety to the cognate part of the aptamer domain. The reporter gene assay presented herein however suggests that *in vitro* binding does not always necessarily correlate with *in vivo* riboswitch regulation. Even though **TT** does not contain a charged middle ring, it induces reporter gene repression more efficiently than **PT**. Moreover, our study shows that riboswitch regulation by artificial activators is dependent on the individual set of metabolic enzymes present in the organism.

Since **TT (1)** is active in *E. coli* and reveals superior activity when compared to pyrithiamine it represents a very promising starting point for developing novel antibacterial compounds that target TPP-riboswitches. Furthermore, our study shows that even being very similar from a structural point of view, **TT** and **PT** engage diverse endogenous mechanisms to attain activity. These findings are of importance for the design and understanding of compounds that require intracellular activation to achieve an effective repression of gene expression.

### Conflict of interest statement

The authors declare that the research was conducted in the absence of any commercial or financial relationships that could be construed as a potential conflict of interest.
