# Supplementary material for: Novel TPP-riboswitch activators bypass metabolic enzyme dependency
Source: Front Chem. 2014 Jul 28;2:53. doi: 10.3389/fchem.2014.00053 (PMC4112796; doi:10.3389/fchem.2014.00053)
Supplement: Supplementary file 1 [file DataSheet1.PDF]

Supplementary information

Novel TPP-riboswitch activators bypass metabolic enzyme dependency

Christina E. Lünse[1], Fraser J. Scott[2], Colin J. Suckling[2,*], and Günter Mayer[1,*]

[1]Life and Medical Sciences Institute, University of Bonn, Gerhard-Domagk-Str. 1, Bonn, Germany

[2]University of Strathclyde, Department of Pure & Applied Chemistry, Glasgow G1 1XL, Scotland

Inventory

Supplementary Table 1
Supplementary Figure 1
Supplementary Figure 2
Supplementary Figure 3
Supplementary Figure 4
Supplementary Figure 5
Supplementary Figure 6
Supplementary Figure 7
Supplementary Figure 8
Supplementary Figure 9
Supplementary Figure 10
Supplementary Figure 11

Supplementary Table 1:

Bacterial Strain	CGSC#	Genotype
DH5αZ1	-	laci^{q}, PN25-tetR, Sp^{R}, deoR, supE44, Δ(lacZYA-argFV169), ϕ80 lacZΔM15, hsdR17(rK- mK+), recA1, endA1, gyrA96, thi-1, relA1
BW25113	7636	Δ(araD-araB)567, ΔlacZ4787(::rrnB-3),λ^{-}, rph-1, Δ(rhaD-rhaB)568, hsdR514
ΔthiQ (JW0065-1)	8367	F-, Δ(araD-araB)567, ΔthiQ773::kan, ΔlacZ4787(::rrnB-3), λ^{-}, rph-1, Δ(rhaD-rhaB)568, hsdR514
ΔthiP (JW0066-1)	8368	F-, Δ(araD-araB)567, ΔthiP774::kan, ΔlacZ4787(::rrnB-3), λ^{-}, rph-1, Δ(rhaD-rhaB)568, hsdR514
ΔtbpA (JW0067-2)	8369	F-, Δ(araD-araB)567, ΔtbpA775::kan, ΔlacZ4787(::rrnB-3), λ^{-}, rph-1, Δ(rhaD-rhaB)568, hsdR514
ΔthiI (JW0413-1)	8580	F-, Δ(araD-araB)567, ΔlacZ4787(::rrnB-3), ΔthiI780::kan, λ^{-}, rph-1, Δ(rhaD-rhaB)568, hsdR514
ΔthiK (JW1092-1)	9034	F-, Δ(araD-araB)567, ΔlacZ4787(::rrnB-3), λ^{-}, ΔthiK768::kan, rph-1, Δ(rhaD-rhaB)568, hsdR514
ΔthiD (JW2090-2)	9701	F-, Δ(araD-araB)567, ΔlacZ4787(::rrnB-3), λ^{-}, ΔthiD728::kan, rph-1, Δ(rhaD-rhaB)568, hsdR514
ΔthiM (JW2091-2)	9702	F-, Δ(araD-araB)567, ΔlacZ4787(::rrnB-3), λ^{-}, ΔthiM729::kan, rph-1, Δ(rhaD-rhaB)568, hsdR514
ΔthiH (JW3953-2)	8311	F-, Δ(araD-araB)567, ΔlacZ4787(::rrnB-3), λ^{-}, rph-1, Δ(rhaD-rhaB)568, ΔthiH760::kan, hsdR514
ΔthiS (JW3955-2)	10847	F-, Δ(araD-araB)567, ΔlacZ4787(::rrnB-3), λ^{-}, rph-1, Δ(rhaD-rhaB)568, ΔthiS762::kan, hsdR514
ΔthiF (JW3956-1)	8312	F-, Δ(araD-araB)567, ΔlacZ4787(::rrnB-3), λ^{-}, rph-1, Δ(rhaD-rhaB)568, ΔthiF763::kan, hsdR514
ΔthiE (JW3957-1)	8313	F-, Δ(araD-araB)567, ΔlacZ4787(::rrnB-3), λ^{-}, rph-1, Δ(rhaD-rhaB)568, ΔthiE764::kan, hsdR514
ΔthiC (JW3958-1)	8314	F-, Δ(araD-araB)567, ΔlacZ4787(::rrnB-3), λ^{-}, rph-1, Δ(rhaD-rhaB)568, ΔthiC765::kan, hsdR514
ΔthiG (JW5549-1)	8316	F-, Δ(araD-araB)567, ΔlacZ4787(::rrnB-3), λ^{-}, rph-1, Δ(rhaD-rhaB)568, ΔthiG761::kan, hsdR514

ΔsufS (JW1670-1)	9419	F-, Δ(araD-araB)567, ΔlacZ4787(::rrnB-3), λ^-, ΔsufS755::kan, rph-1, Δ(rhaD-rhaB)568, hsdR514
ΔiscS (JW2514-4)	10002	F-, Δ(araD-araB)567, ΔlacZ4787(::rrnB-3), λ^-, ΔiscS776::kan, rph-1, Δ(rhaD-rhaB)568, hsdR514

Supplementary Table 1: This table lists bacterial strains used in this study and indicates their genomic mutations. For detailed explanation of abbreviations and genomic alterations refer to the CGSC website (http://cgsc.biology.yale.edu/)

Supplementary Figure 1

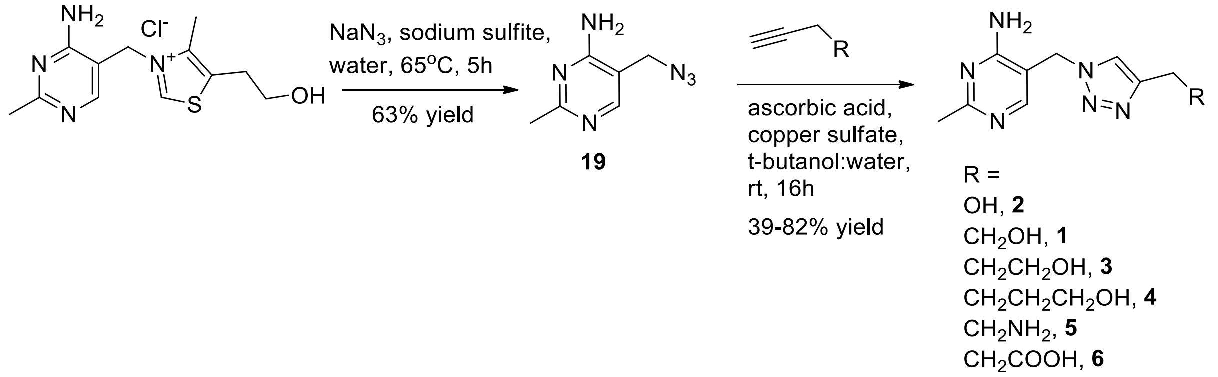

Supplementary Figure 1: Synthesis scheme of compounds **1-6** was achieved using a "click" chemistry approach utilizing a common azide intermediate, **19**. This was obtained from thiamine itself in a substitution reaction with sodium azide which has been previously reported by Erixon *et al*. (Erixon, Dabalos et al. 2007; Erixon, Dabalos et al. 2008). Compound **19** was then reacted with the appropriate substituted alkyne to generate the small library of six thiamine derivatives.

Supplementary Figure 2:

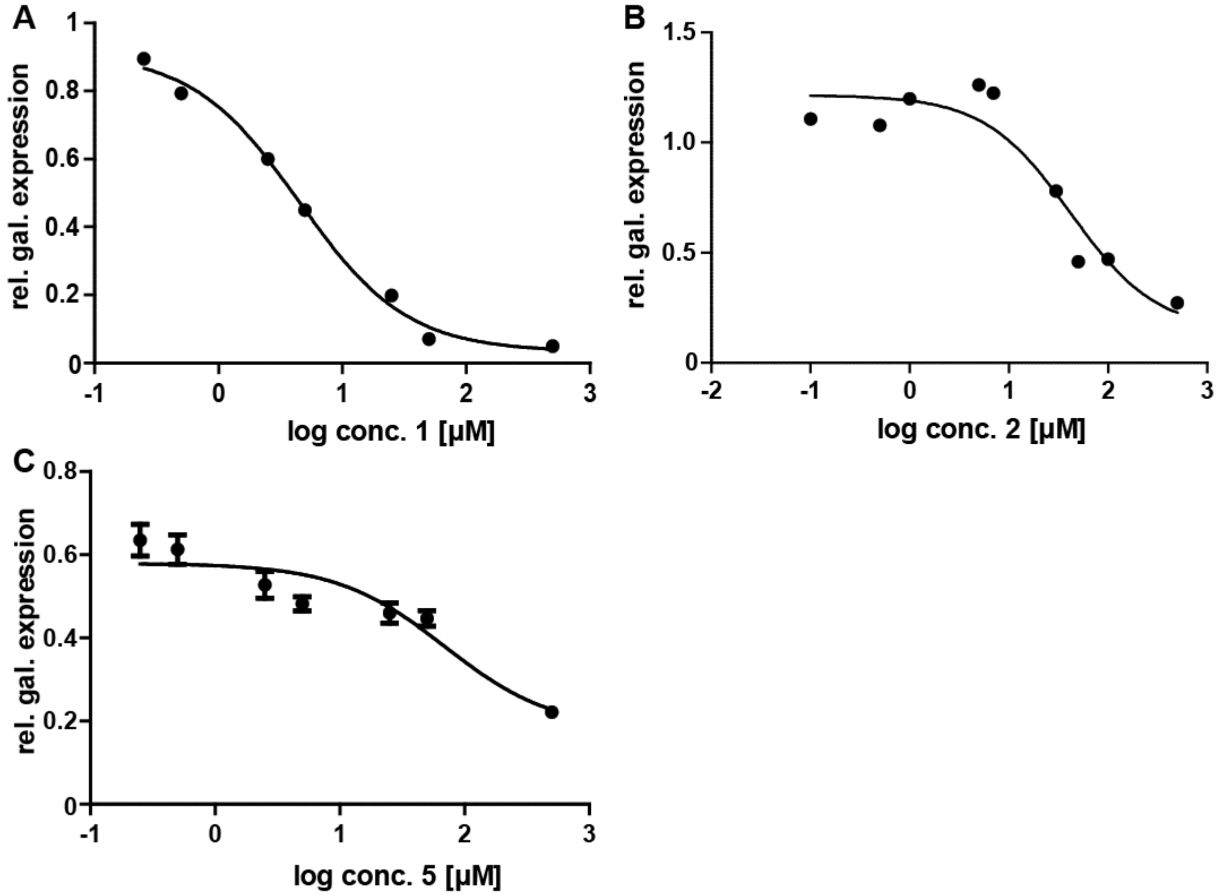

Supplementary Figure 2: Inhibitory concentration curves obtained by monitoring β-galactosidase expression in DH5αZ1 cells (Diederich, Rasmussen et al. 1992; Lutz and Bujard 1997) with increasing compound concentrations (0.01-500µM) for compound **1** (**A**), **2** (B) and **5** (C) are shown. Each experiment, of which one exemplary curve is depicted here, was measured in duplicates and repeated at least two times. Mean and 95% confidence intervals of these measurements are shown in Table 1.

Supplementary Figure 3:

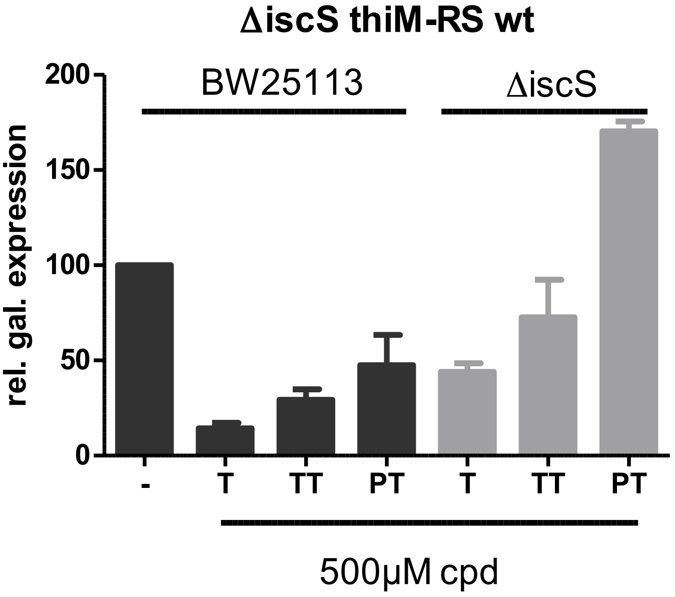

Supplementary Figure 3: Relative β-galactosidase expression in the presence of thiamine, TT and PT in wild type and ΔiscS *E. coli* was monitored. The deletion of iscS renders bacteria less susceptible to thiamine, **TT** or **PT**. Additionally, decelerated growth of the deletion strain was observed.

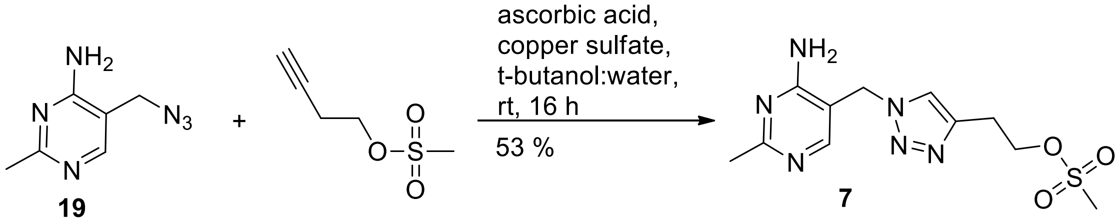

19 **7**

Supplementary Figure 4: Compound **7** was prepared in a similar fashion to compounds **1-6** by a 'click' reaction of **19** with the appropriate substituted alkyne.

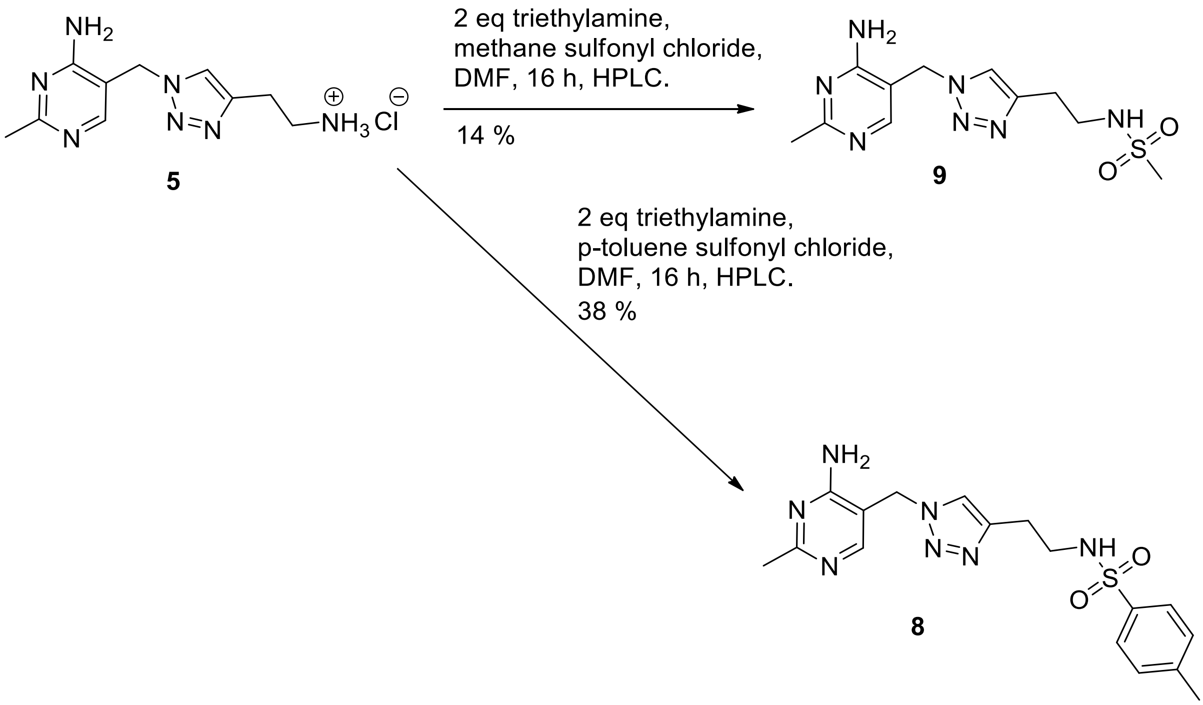

Supplementary Figure 5: Compounds **8** and **9** were prepared by amide coupling with the appropriate acid chloride.

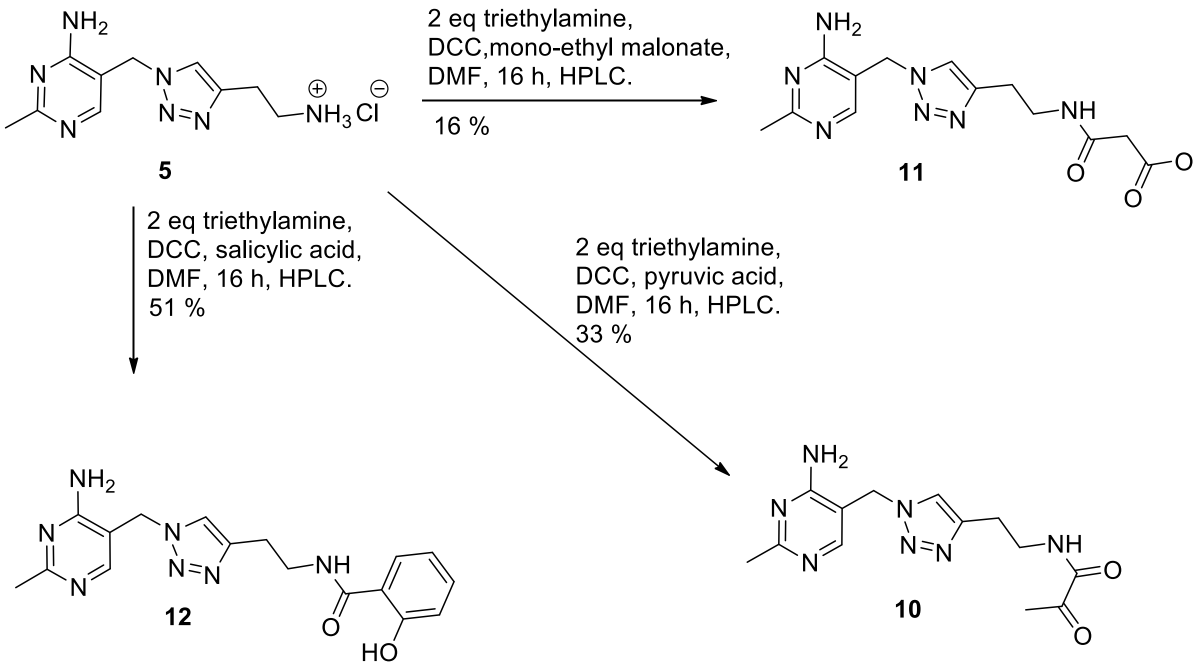

Supplementary Figure 6: Compounds **10**, **11** and **12** were prepared by amide coupling with the appropriate carboxylic acid.

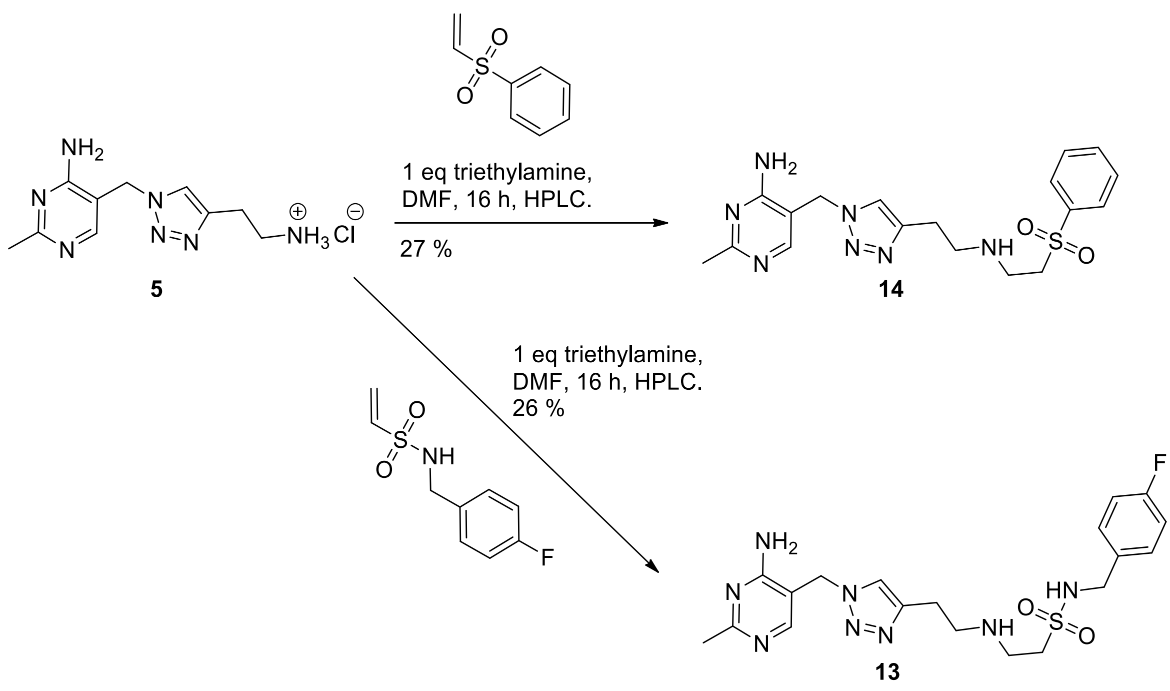

Supplementary Figure 7: Compounds **13** and **14** was prepared by the reaction of **5** with the appropriate Michael acceptor.

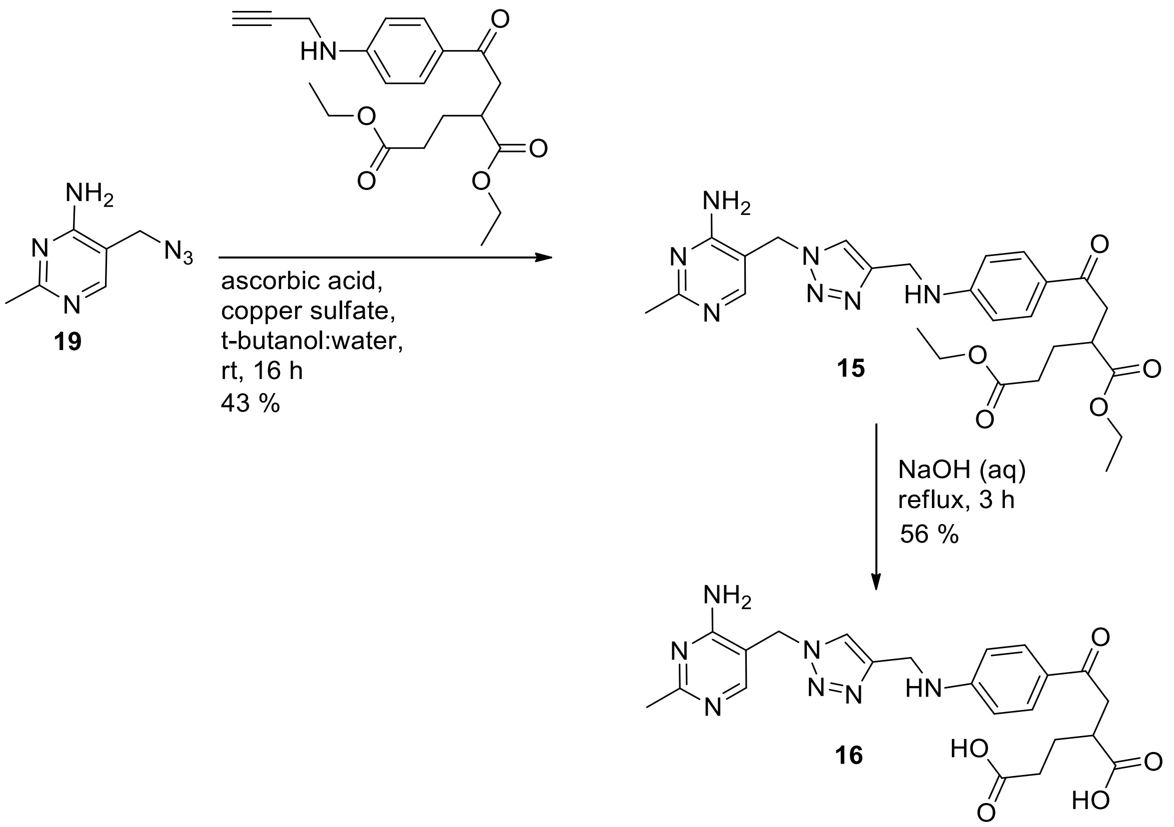

Supplementary Figure 8: Compound **15** was prepared in a similar fashion to compounds **1-6** by a 'click' reaction of **19** with the appropriate substituted alkyne. This was then hydrolyzed to obtain **16**.

Supplementary Figure 9:

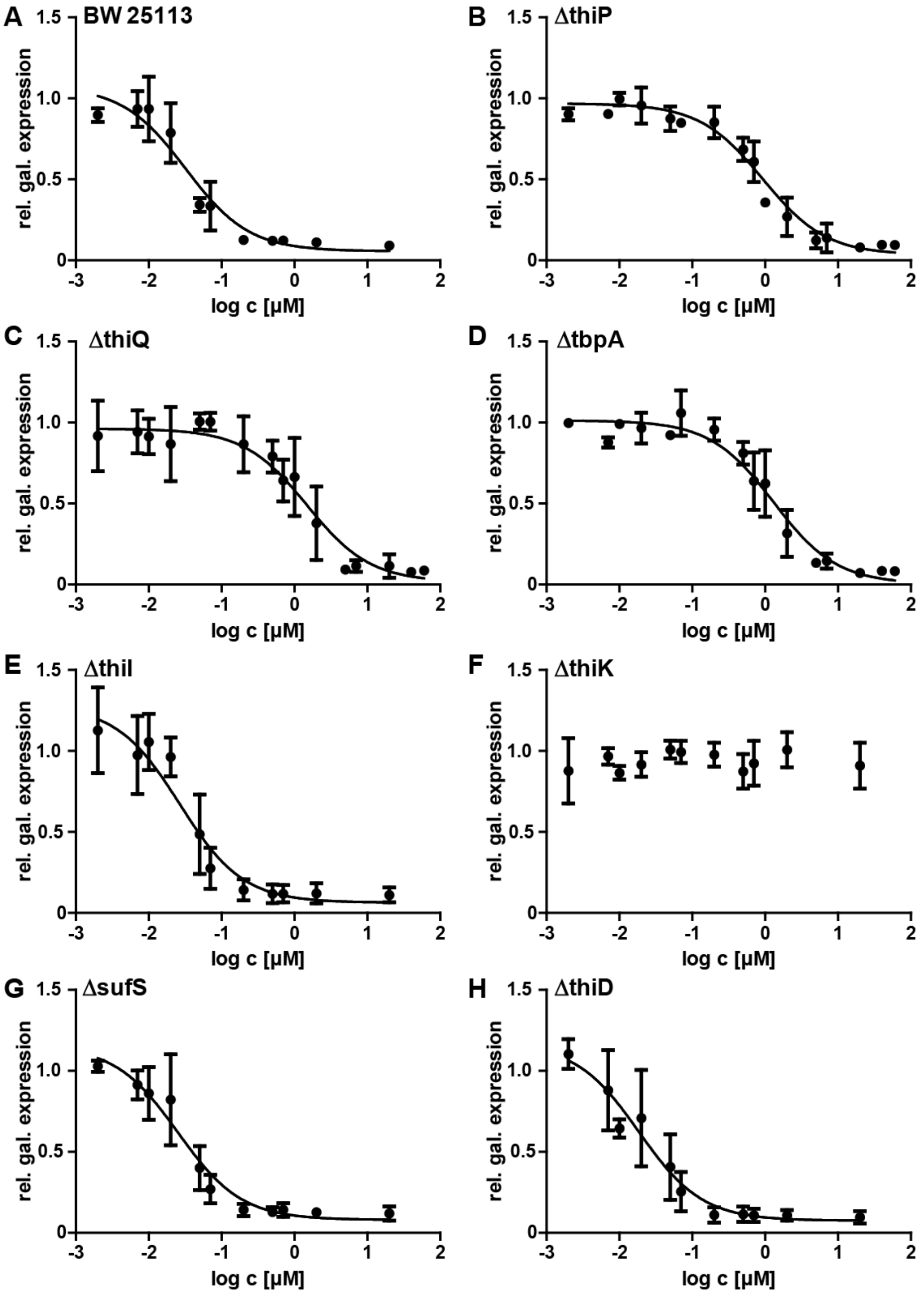

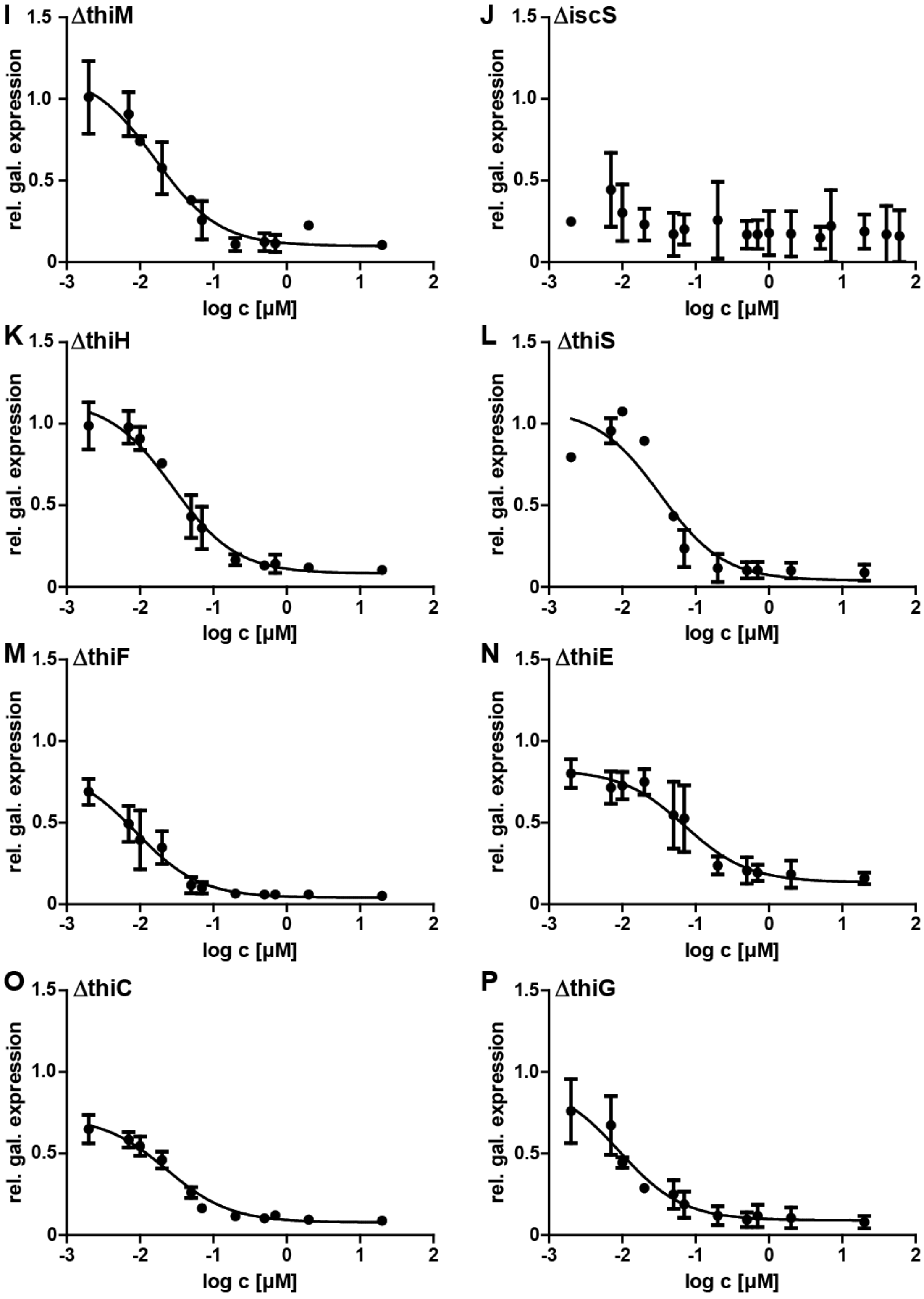

Supplementary Figure 9: β-galactosidase expression in the presence of increasing thiamine concentrations for IC_{50} determinations. Investigation of Keio deletion strains (A-P) containing the wildtype thiM riboswitch variant.

Supplementary Figure 10:

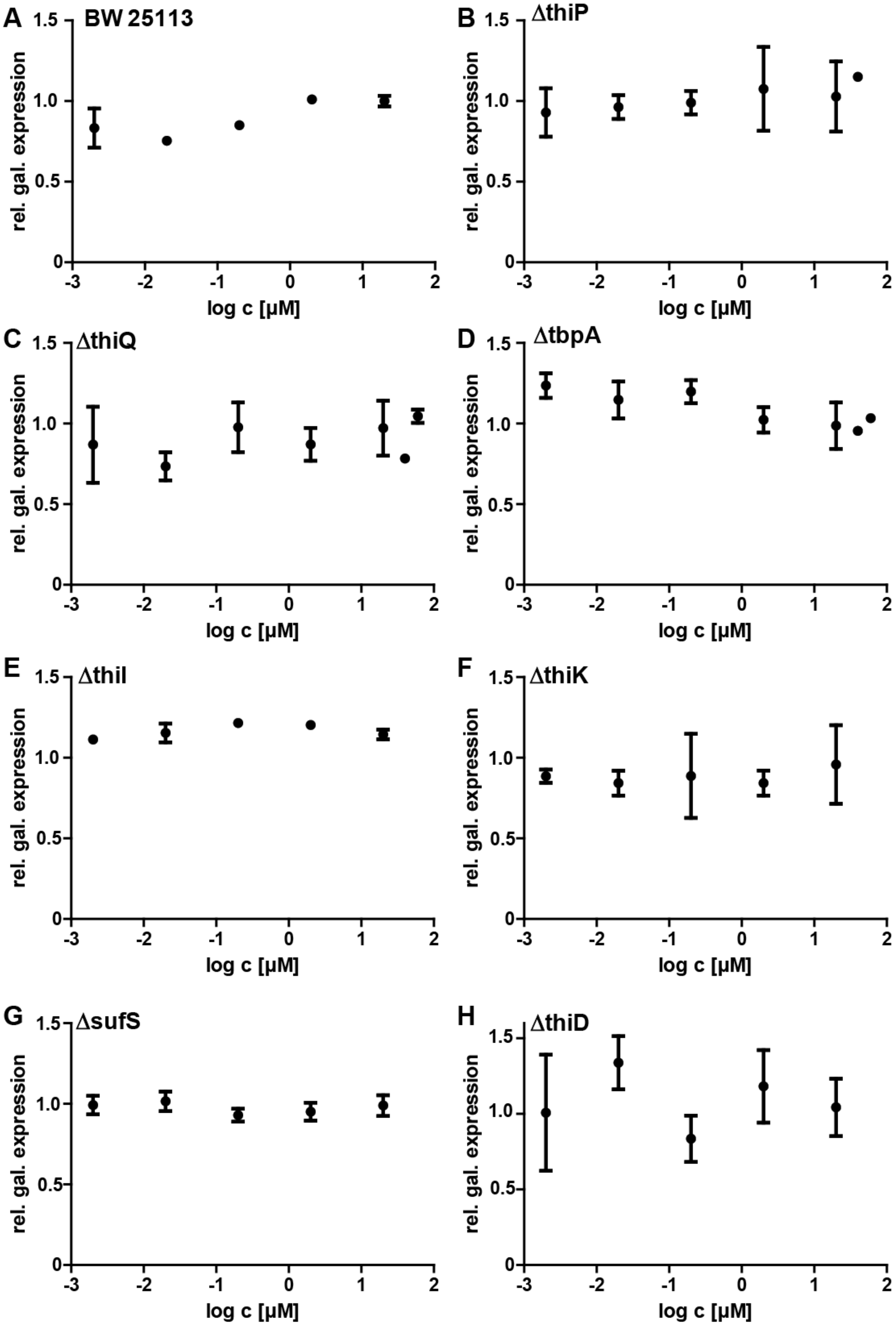

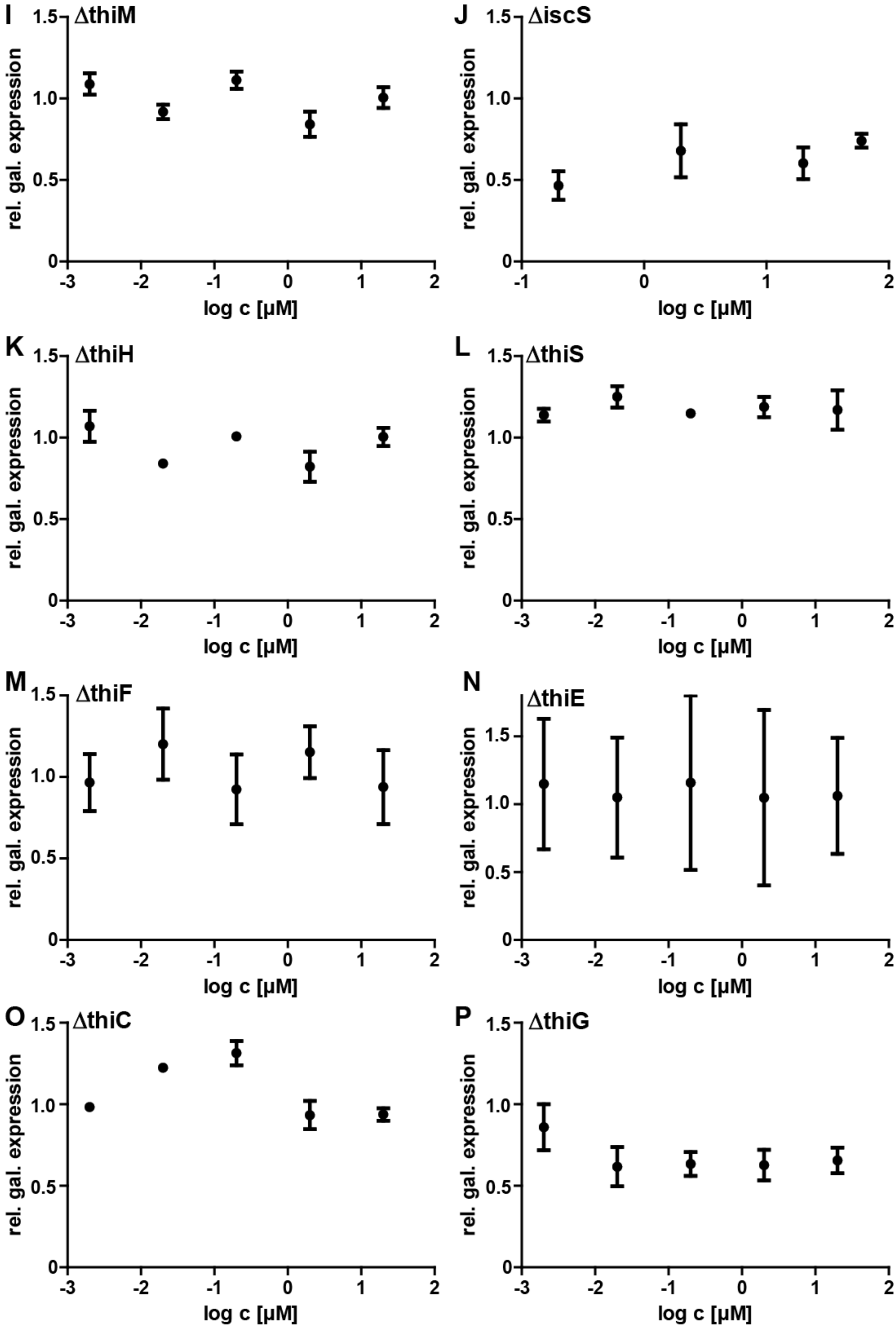

Supplementary Figure 10: β-galactosidase expression in the presence of increasing thiamine concentrations for IC_{50} determinations. Investigation of Keio deletion strains (A-P) containing the mutated thiM riboswitch variant.

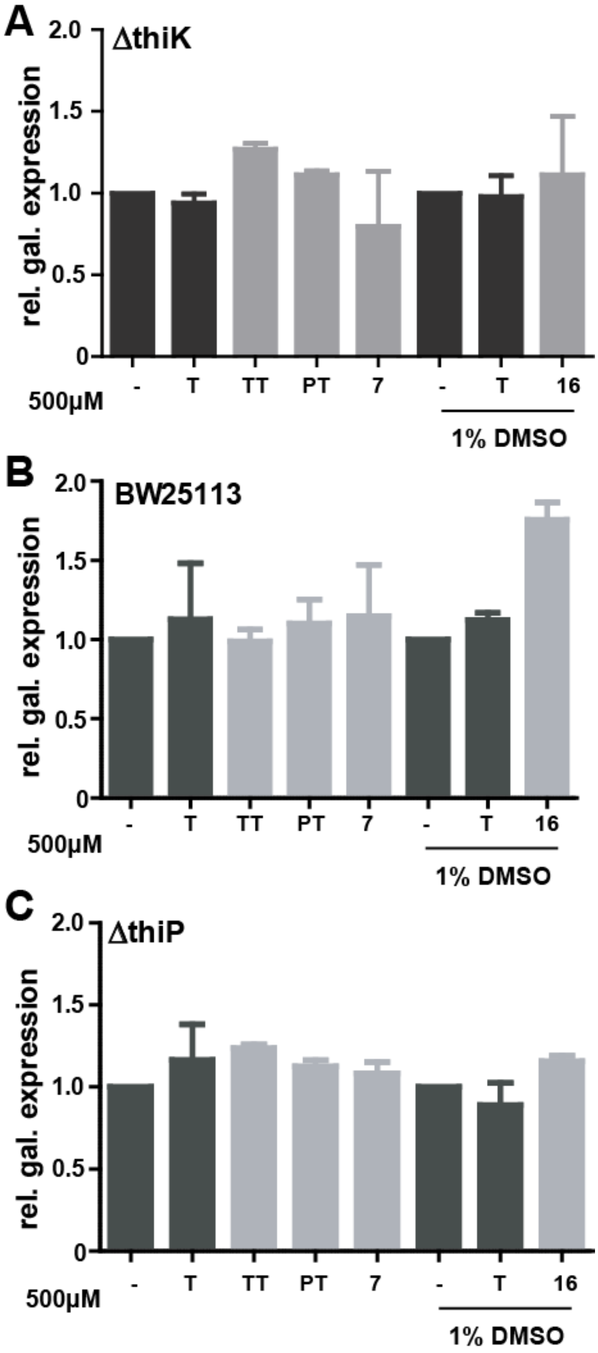

Supplementary Figure 11: β-galactosidase expression in the presence of thiamine, triazolethiamine (TT), pyrithiamine (PT) and compounds 7 and 16. Investigation of Keio strains (A-C) containing the mutated thiM riboswitch variant (thiM-Mu).

References:

Diederich, L., L. J. Rasmussen, et al. (1992). "New cloning vectors for integration in the lambda attachment site attB of the Escherichia coli chromosome." Plasmid **28**(1): 14-24.

Erixon, K. M., C. L. Dabalos, et al. (2007). "Inhibition of pyruvate decarboxylase from Z. mobilis by novel analogues of thiamine pyrophosphate: investigating pyrophosphate mimics." Chemical communications(9): 960-962.

Erixon, K. M., C. L. Dabalos, et al. (2008). "Synthesis and biological evaluation of pyrophosphate mimics of thiamine pyrophosphate based on a triazole scaffold." Organic & biomolecular chemistry **6**(19): 3561-3572.

Lutz, R. and H. Bujard (1997). "Independent and tight regulation of transcriptional units in Escherichia coli via the LacR/O, the TetR/O and AraC/I1-I2 regulatory elements." Nucleic Acids Res **25**(6): 1203-1210.
